# Curative Effect of 18β-Glycyrrhetinic Acid in Experimental Visceral Leishmaniasis Depends on Phosphatase-Dependent Modulation of Cellular MAP Kinases

**DOI:** 10.1371/journal.pone.0029062

**Published:** 2011-12-14

**Authors:** Anindita Ukil, Susanta Kar, Supriya Srivastav, Kuntal Ghosh, Pijush K. Das

**Affiliations:** 1 Department of Biochemistry, Calcutta University, Kolkata, India; 2 Molecular Cell Biology Laboratory, Infectious Diseases and Immunology Division, Indian Institute of Chemical Biology, Kolkata, India; University Paris Sud, France

## Abstract

We earlier showed that 18β-glycyrrhetinic acid (GRA), a pentacyclic triterpenoid from licorice root, could completely cure visceral leishmaniasis in BALB/c mouse model. This was associated with induction of nitric oxide and proinflammatory cytokine production through the up regulation of NF-κB. In the present study we tried to decipher the underlying cellular mechanisms of the curative effect of GRA. Analysis of MAP kinase pathways revealed that GRA caused strong activation of p38 and to a lesser extent, ERK in bone marrow-derived macrophages (BMDM). Almost complete abrogation of GRA-induced cytokine production in presence of specific inhibitors of p38 and ERK1/2 confirmed the involvement of these MAP kinases in GRA-mediated responses. GRA induced mitogen- and stress-activated protein kinase (MSK1) activity in a time-dependent manner suggested that GRA-mediated NF-κB transactivation is mediated by p38, ERK and MSK1 pathway. As kinase/phosphatase balance plays an important role in modulating infection, the effect of GRA on MAPK directed phosphatases (MKP) was studied. GRA markedly reduced the expression and activities of three phosphatases, MKP1, MKP3 and protein phosphatase 2A (PP2A) along with a substantial reduction of p38 and ERK dephosphorylation in infected BMDM. Similarly in the *in vivo* situation, GRA treatment of *L. donovani*-infected BALB/c mice caused marked reduction of spleen parasite burden associated with concomitant decrease of individual phosphatase levels. However, activation of kinases also played an important role as the protective effect of GRA was significantly abrogated by pharmacological inhibition of p38 and ERK pathway. Curative effect of GRA may, therefore, be associated with restoration of proper cellular kinase/phosphatase balance, rather than modulation of either kinases or phosphatases.

## Introduction

Visceral leishmaniasis (VL), caused by the parasite *Leishmania donovani*, is a major health problem with significant morbidity and mortality worldwide [1]. In order to survive successfully inside the macrophages, *Leishmania* employs several strategies to macrophage defence mechanism which subsequently culminates in defective immune subvert response [2]. Most of the drugs used for the therapy of this disease, such as antimonials as first-line and amphotericin B and pentamidine as second-line drugs, have serious side effects that limit their clinical application [3]. Therefore immunomodulators, with our present day knowledge of the immune system, are creating new opportunities for the development of immunomodulatory therapies [4], [5] in VL.

During the last decade, a large number of dietary components have been evaluated as potential chemopreventive agents [6]. However, limited scientific evidence regarding the effectiveness of these natural derivatives, in conjunction with a lack of mechanistic understanding of their actions, has prevented their incorporation into the mainstream of medical care. Licorice (*Glycyrrhizza glabra L.*) and its main water soluble component 18β-glycyrrhetinic acid (GRA) have been widely used as a food flavouring and sweetening agent in diet [7]. GRA has a long history of medicinal use and is known to exhibit a variety of pharmacological effects including anti-tumor, anti-hepatotoxic, and immunomodulatory activities [8]–[11]. Immunomodulatory effects of GRA may be mediated by the favourable expression of nitric oxide (NO) and proinflammatory cytokines from macrophages [12]–[14]. The major transcription factor responsible for GRA-mediated immunomodulatory effect and NO production is NF-κB [12]. We have previously demonstrated that GRA triggers curative Th1 response and NO up-regulation in experimental VL by activating NF-κB [15]. But the molecular signaling pathway, which controls the activation of NF-κB is yet to be explored.

A number of studies have indicated that activation of NF-κB correlates with the increased activation of the mitogen-activated protein kinase (MAPK) pathway [16], [17]. An earlier report shows that viscerotropic *L. donovani* parasites fail to induce the activation of the MAPKs in naive murine bone marrow-derived macrophages (BMDM) [18]. *L donovani* also modulated the TLR2-stimulated MAPK pathway by suppressing MAPK P38 phosphorylation [19]. Recently we have shown the importance of MEK-ERK and JAK-STAT signaling pathways in down regulating *Leishmania* infection using cystatin as a model anti-leishmanial compound [20]. A few scattered evidences have also been found in support of the defective TLR response during *Leishmania* infection [21]–[23]. It is, therefore, quite obvious that MAPK pathway holds an important role among several other strategies which *Leishmania* deploys to undermine host response in order to survive and replicate within macrophages.

Kinase mediated phosphorylation plays an important role in macrophage activation, but uncontrolled activation could be detrimental for the cell. It is necessary for the cells that both the protein kinases and phosphatases maintain their physiological balance to sustain a normal regulation of events. Recent studies have demonstrated that protein phosphatases may serve as pivotal feedback control regulators in the innate immune response during *Leishmania* infection [24], [25]. A number of reports have demonstrated the important role played by SHP-1 in establishing and augmenting the course of visceral infection [24], [26]. SHP-1 dephosphorylates a number of vital kinases and SHP-1-deficient mice showed a much stronger proinflammatory response against *Leishmania* infection compared to wild-type mice. Induction of SHP-1 by *Leishmania* is essential for inhibition of NO generation and this occurs through the inactivation of JAK2 and ERK1/2, and transcription factors NF-κB and AP-1 [24]. Recently, we have identified three MAPK directed phosphatases which are upregulated during visceral infection and inhibition of these phosphatases in *L. donovani*-infected mice shifted the cytokine balance in favour of the host [27]. The present study is aimed to uncover the critical events in the cellular signaling network involving kinase/phosphatase balance during *Leishmania* infection by GRA, a compound of herbal origin, which is safe, nontoxic and therefore could be used as potential drug for diseases where till date no satisfactory treatment is available.

## Results

### Therapeutic effects of GRA through the upregulation of NO and Th1 cytokines

**Figure 1:**
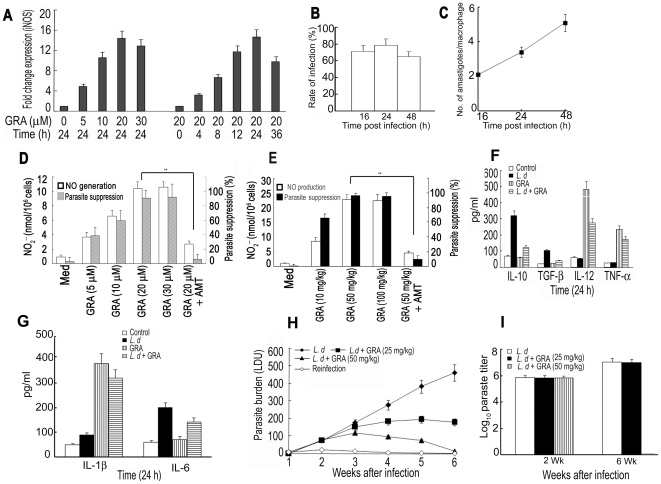
Antileishmanial activity of GRA. (**A**) Dose- and time-dependent expression of iNOS by Taqman analysis of the mRNA transcript in BMDM stimulated with GRA. mRNA levels were normalized to β-actin and expressed as a -fold change compared with control. (**B**) Percentages of infected cells and (**C**) number of parasites per macrophage were counted at various time periods following infection. (**D**) Effect of GRA on NO production (solid bar) and parasite suppression (open bar) in BMDM in presence or absence of AMT (10 µM). (**E**) Generation of NO (solid bar) and parasite suppression (open bar) in peritoneal macrophages isolated from mice which received GRA (10, 50 and 100 mg/kg) i.p. for 3 times 5 days apart. Macrophages were harvested 10 h after the last injection and infected with *L. donovani*. After 4 h infection and 20 h of incubation, the percentage of parasite suppression and the amount of NO_2_^−^ in the medium were determined. (**F**) and (**G**) Levels of IL-12, TNF-α, IL-10 ,TGF-β, IL-6 and IL-1β in culture supernatants from infected and GRA (20 µM)-treated BMDM were determined by ELISA. (**H**) Mice were administered i.v. with 10^7^ promastigotes, and after 10 days of infection, were treated with GRA (25 or 50/mg/kg) i.p. 3 times 5 days apart. Spleen parasite burdens were determined 6 wk after infection and are expressed as LDU ± SD. (**I**) Spleen parasite burden determined 2 and 6 wk following infection measured by limiting dilution assay expressed as log_10_ parasite burden ± SD. Cultures were set in triplicate and experiments were done a minimum of three times. Animal experiments were done with five animals/group and the results are representative of three independent experiments. Data represent the mean ± SD. ***p<0.001; Student's t-test.

Treatment of BMDM with GRA resulted in increased iNOS mRNA expression in a dose- and time-dependent manner (**Figure 1A**). Maximum expression (14.4-fold) was obtained at 24 h with 20 µM GRA. Maximum nitrite production (10.4 nmol/10^6^ cells) as well as maximum inhibition of intracellular amastigote count (91.3% suppression) was also observed in cells treated with 20 µM GRA for 24 h (**Figure 1D**). *In vivo* administration of GRA resulted in much higher NO generation and antileishmanial activity. Peritoneal macrophages from GRA (50 mg/kg/day)-administered mice exhibited 22.8±1.6 nmol/10^6^ cells of NO_2_^−^ and 97.7% parasite suppression compared to 10.4 nmol/10^6^ cells of NO_2_^−^ and 91.3% suppression from their *in vitro* counterpart (**Figure 1E**). Nitrite production as well as antileishmanial activity of both *in vitro* and *ex vivo* effects of GRA were significantly abrograted by 2-amino-5,6-dihydro-6-methyl-4H-1,3-thiazine (AMT), an inhibitor of iNOS, suggesting that the antileishmanial effect of GRA may be correlated with increased production of NO (**Figure 1D and 1E**). GRA treatment of *L. donovani*-infected BMDM potentiated Th1 type immunity, evident from a significant rise in IL-12 (5.2-fold), TNF-α (5.8-fold) and IL-1β (3.56 fold) production with concomitant decrease of IL-10 (78%) and TGF-β (75%), signature Th2 cytokines, compared to infected BMDM (**Figure 1F and G**). However, the level of IL-6, which was increased after *Leishmania* infection, was not altered by GRA treatment (**Figure 1G**). This is not surprising as IL-6 has been reported to exert an early, suppressive effect on host defense by restraining Th1-cell-type antileishmanial responses [28]. When tested in a mouse model of VL, a dose of 50 mg/kg/day given i.p. 3 times 5 days apart starting at 10 days after infection resulted in possibly complete elimination of spleen parasite burden (**Figure 1H**) at 6 wk after infection. Absence of parasites in the spleen of treated animals was further confirmed by culturing spleen specimens in transformation medium for 4 days at 22°C and limiting dilution assay for 2-wk and 6-wk sample (**Figure 1I**). The protective effect of GRA was documented by reinfecting cured animals 8 wk after infection (data not shown). Very little increase in the spleen parasite burden suggested that along with macrophage activating immunomodulatory properties, GRA also imparted significant protection against further infection.

### GRA activates NF-κB cascade in *L. donovani* infected mouse spleen

**Figure 2:**
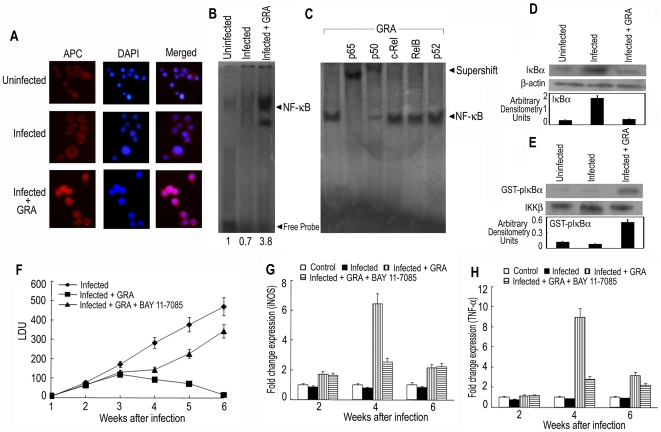
Involvement of NF-κB in GRA-mediated anti-leishmanial response. (**A**) *L. donovani*-infected and GRA (20 µM)-treated BMDM were stained with anti-p65 monoclonal antibody and APC-conjugated secondary antibody. Nuclei were stained with DAPI and cells were analyzed under fluorescence microscope as described in Materials and Methods. (**B** and **C**) *L. donovani*-infected mice were treated with GRA (50 mg/kg) for 3 times 5 days apart starting at 10 days after infection and splenocytes were isolated 3 days after last treatment. Labeled NF-κB probe was incubated with nuclear extracts of splenocytes and EMSA was performed (**B**). The bands were analyzed densitometrically and –fold changes are indicated. (**C**) For supershift assay, nuclear extracts from splenocytes were incubated with antibodies against individual components of NF-κB complex for 30 min. The results are representative of one of three separate experiments. (**D**) Western blot analysis shows a depletion of IκBα protein in splenocytes of GRA-treated mice as opposed to increased preservation in infected group. (**E**) IKKβ was immunoprecipitated from splenocyte cell lysate and IKK activity was assayed using GST-IκBα as substrate, and GST-phosphorylated IκBα was visualized by autoradiography. Relative amount of IKKβ was determined by Western blots. *L. donovani*-infected mice were treated either with GRA (50 mg/kg) as described in the legend of **Figure 1E** or with GRA plus BAY11-7085 (5 mg/kg/day) 3 times weekly for 4 wk starting at 2 wk after infection. Parasite burden in spleen (**F**) mRNA expression of iNOS (**G**) and the level of TNF-α (**H**) in the splenocytes were determined. Results are representative of three individual experiments and the error bars represent mean ± SD (n = 3). ***p<0.001; Student's t-test.

We earlier demonstrated that in cultured macrophages, GRA activates NF-κB signaling pathway at some level upstream of IKK [15]. In the present study, these experiments were done in primary cells (mouse BMDM), which might be more relevant cellular context to parallel murine model of visceral leishmaniasis. Induction of IKK/NF-κB activation is also operative in primary cells like BMDM as reflected in the nuclear translocation of NF-κB p65 subunit judged by fluorescence microscopy. In unstimulated and *L. donovani*-infected macrophages, the signal for p65 was distributed throughout the cell but did not colocalize with DAPI-stained nuclei indicating its cytosolic localization (**Figure 2A**). On the contrary, GRA stimulation resulted in induction in the nuclear import of p65 as evident by pronouncedly enhanced nuclear colocalization within 2 h of treatment (**Figure 2A**). In order to ascertain its status *in vivo*, we examined the relative abundance, subcellular distribution and functional activity of NF-κB in infected mice. A curative dose of 50 mg/kg/day of GRA were given 3 times 5 days apart starting at 10 days after infection and splenocytes were isolated 3 days after the final treatment. Binding to oligonucleotides containing a consensus NF-κB response element was markedly increased (5.4-fold) in GRA-treated infected mice compared to infected control (**Figure 2B**). To identify the specific NF-κB subunits that comprise the NF-κB signal detected by EMSA, supershift assay was performed with specific antibodies against p50, p52, p65, c-Rel and Rel B. Both p65 and p50 antibodies caused a significant shift of the entire signal, whereas anti-p52, and anti-c-Rel and anti-Rel B antibodies did not have any effect (**Figure 2C**). Western blot analysis of the cytoplasmic extract prepared from splenocytes revealed that IκBα protein levels were greatly depleted (95.1%) in GRA-treated infected animals indicating that NF-κB activation occurred via IκBα degradation (**Figure 2D**). Since IκBα is thought to be phosphorylated by the IKKβ component of the IKK multiprotein complex, IKKβ was immunoprecipitated from splenocytes and tested for the ability to phosphorylate GST-IκBα substrate. As shown in **Figure 2E**, GRA treatment strongly induced IKKβ activity (6.25-fold) in the spleen of infected mice. Western blotting for IKKβ was performed as a loading control and showed that similar amounts of IKKβ were immunoprecipitated from the splenocyte extracts. All these results suggest that activation of NF-κB is induced in the spleen of *L. donovani*-infected mice by treatment with GRA. In order to ascertain whether the induction of NF-κB activation was directly correlated with the therapeutic effect of GRA, we used BAY 11-7085, which is known to attenuate both the phosphorylation of IκBα and NF-κB activity *in vivo* [29]. As shown in **Figure 2F**, a dose of 5 mg/kg/day of BAY 11-7085 given 3 times weekly for 4 wk starting at 2 wk after infection could significantly reverse GRA-mediated suppression of spleen parasite burden (70.2% reduction) and GRA-mediated increase of iNOS (71.7% reduction) (**Figure 2G**) and TNF-α (77.6% reduction) (**Figure 2H**) expression, as revealed by Real time PCR analysis, stressing further that the therapeutic effect of GRA may be attributed to the activation of NF-κB pathway in *L. donovani*-infected mice.

### GRA activates MAPK pathways

**Figure 3:**
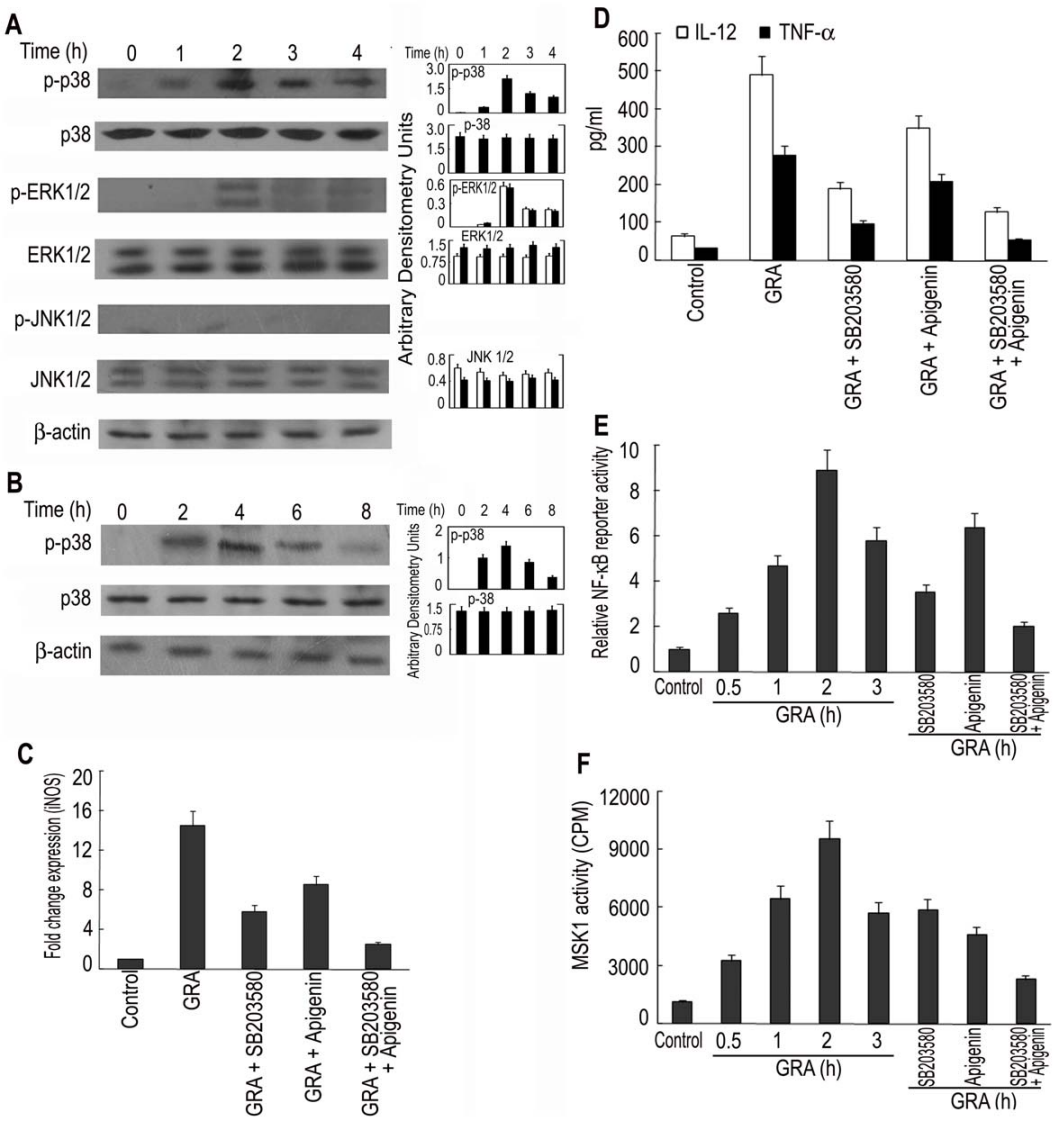
Effect of GRA on the regulation of iNOS and Th1 cytokine by MAPK activation. (**A**) BMDM were treated with GRA (20 µM) for different time periods. The expression and phosphorylation of MAPKs were detected by Western blotting. (**B**) *L. donovani*-infected BMDM were treated with GRA (20 µM) for various time periods and the level of phosphorylated p38 was measured by Western blotting. (**C** and **D**) BMDM were treated (1 h) with either SB203580 (30 µM) or apigenin (40 µM) or both before stimulation with GRA (24 h). iNOS expression (**C**) by Taqman analysis and levels of IL-12 and TNF-α (**D**) by ELISA were determined. (**E**) RAW 264.7 cells were transiently transfected using Lipofectamine reagent with 1 µg of NF-κB luciferase reporter vector along with 0.5 µg pcMV-βgal. After 24 h, cells were stimulated with GRA (20 µM) for different time periods. In a separate set of experiment, transfected cells were pre-incubated with either SB203580 (30 µM) or apigenin (40 µM) or both for 1 h before stimulation with GRA (12 h). Cells were lysed and processed for luciferase activity. (**F**) BMDM were treated with GRA (20 µM) for different time periods or pre-incubated with either SB203580 (30 µM) or apigenin (40 µM) or both for 1 h before stimulation with GRA (2 h). Cell lysates were then immunoprecipitated with anti-MSK1 Ab and MSK1 activity was assayed using CREBTIDE as substrate. Bands were analyzed densitometrically. Error bars represent mean ± SD (n = 3). The data shown are representative of three independent experiments. ns, not significant; *p<0.05, **p<0.01, ***p<0.001; Student's t-test.

To understand the molecular mechanism of GRA-mediated activation of macrophages, next we investigated the MAPK signaling pathways, which are known to play a major role in iNOS and pro-inflammatory cytokine production. Kinetic study with BMDM showed that GRA (20 µM) markedly induced the phosphorylation of p38 at 2 h, and the level remained elevated as studied up to 4 h (**Figure 3A**). GRA also activated ERK1/2, although to a lesser extent as compared to p38, but no induction of p46 and p54 JNK was observed (**Figure 3A**). Kinetic analysis of GRA-treated infected macrophages showed similar pattern with lesser induction and slower kinetics in infected cells (**Figure 3B**). Preincubation of cells for 1 h before GRA stimulation with specific p38 and ERK1/2 inhibitors, SB203580 and apigenin, resulted in dose-dependent reduction of iNOS mRNA expression. A maximum reduction of 64.2% and 44.1% was obtained at the highest concentration of SB203580 (30 µM) and apigenin (40 µM) respectively (**Figure 3C**). Whereas the selective blockage of the p38 pathway markedly inhibited GRA-induced IL-12 and TNF-α expression (71.1% and 73.9% respectively), the effect was minimal in case of ERK1/2 inhibitor (32.9% and 28.8% respectively) (**Figure 3D**). The role of p38 therefore is probably more important in the modulation of GRA-mediated Th1 response. Next we wanted to determine whether NF-κB activation was also under the control of this signaling cascade. Cell exposure to SB203580 resulted in significant decrease (68.2%) of GRA-inducible NF-κB luciferase reporter activity, but a lesser decrease (32.1%) was found in case of apigenin (40 µM) (**Figure 3E**). However, a combination of both inhibitors greatly inhibited luciferase gene expression (87.1%) (**Figure 3E**), suggesting that ERK and p38 MAPK activation are critical in sustaining GRA mediated NF-κB activation. This observation prompted us to investigate the role of MSK1, which is a downstream target of both p38 and ERK and phosphorylates transcription factors. These two MAPKs are reported to activate MSK1 in inflammatory conditions in cell lines like HEK293 and L929sA [30]. GRA could markedly activate MSK1 activity, which was partially inhibited by pretreatment with either SB203580 (59.2% reduction) or apigenin (44.1% reduction) (**Figure 3F**). A combination of both greatly reduced (86.1%) MSK1 activation suggesting that p38 MAPK and ERK act synergistically to activate MSK1 (**Figure 3F**). Taken together, these results suggest that p38/MSK1 and ERK1/2/MSK1-mediated NF-κB activation are necessary for the induction of iNOS and Th1 cytokine expression in response to GRA.

### GRA negatively regulates expression of MAPK directed phosphatases

**Figure 4:**
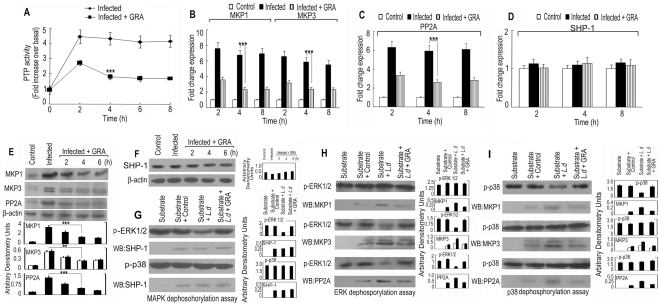
Effect of GRA on cellular kinase/phosphatase balance. BMDM were infected with *L. donovani* promastigotes and treated with GRA (20 µM) for various time periods. (**A**) Induction of PTP activity was measured using a PTP assay kit. Results are expressed as the relative increase (n-fold) over PTP activity in control cells. Expression of MKP1, MKP3 (**B**), PP2A (**C**) and SHP-1 (**D**) at mRNA and at protein level (**E and F**) were determined by Real time PCR and immunoblot analysis respectively. SHP-1, MKP1, MKP3 and PP2A were immunoprecipitated from infected and GRA (20 µM, 4 h)-treated macrophages and assayed for dephosphorylation activity using either recombinant p-ERK or p-p38 as substrate and visualizing by immunoblotting with anti-p-ERK (**G and H**) and anti-p-p38 (**I**) antibody. The amount of individual phosphatases was measured by stripping the blot and reprobing with antibodies against SHP-1, MKP1, MKP3 or PP2A. Results are representative of three individual experiments. Data represent the mean ± SD, n = 3. ***p<0.001; Student's t-test.

Macrophage's signal transduction mechanisms are usually triggered through a series of kinases by protein phosphorylation during infection and are generally counter regulated by various phosphatases, unfortunately sometimes manipulated by the invading pathogens. To understand whether MAP kinase activation by GRA could be an after-effect of down regulation of phosphatase activity, we first checked the total PTP activity in infected and infected GRA-treated macrophages. *L. donovani* infection induced a time dependent increase in PTP activity (4.5-fold at 2 h after infection), which was significantly decreased by GRA treatment (maximum reduction of 75.8% at 4 h) (**Figure 4A**). We then concentrated on the effect of GRA on MAPK directed phosphatases. We have recently documented that three MAPK directed phosphatases, namely MKP1, MKP3 and PP2A play a significant role in visceral infection by augmenting disease progressing Th2 cytokine response [27]. Real time PCR analysis with Taqman probes in L. *donovani*-infected BMMs showed a significant rise in the expression of all three phosphatases (7.6, 6.6- and 6.3-fold increase for MKP1, MKP3 and PP2A respectively) compared to control at 2 h post infection (**Figure 4B and 4C**). On the contrary, treatment with GRA caused a significant decrease in the expression of all three phosphatases at both mRNA (76.2%, 71.9% and 66.8% decrease for MKP1, MKP3 and PP2A respectively) (**Figure 4B and 4C**) and protein (67.6%, 72.1% and 74.2% decrease for MKP1, MKP3 and PP2A respectively) level at 4 h after treatment (**Figure 4E**). Unlike these phosphatases, the expression of SHP-1 remained unchanged both at mRNA and protein level after *L.donovani* infection (**Figure 4D and 4F**), but its activity was increased as evidenced by dephosphorylation assay. To ascertain whether GRA could modulate the infection-induced activation of phosphatases, their activities were assayed using recombinant p-ERK and p-p38 substrates. Dephosphorylation of substrates demonstrated that among the infection-induced phosphatases, pERK serves a better substrate for SHP-1, MKP3 and PP2A (58.6%, 68.3% and 77.9% decrease in phosphorylation for SHP-1, MKP3 and PP2A respectively) (**Figure 4G and 4H**) whereas MKP1 has a preference for p-p38 (60.2% decrease in phosphorylation for MKP1) (**Figure 4G and 4I**). As expected, all phosphatases from GRA-treated infected macrophages showed much lesser dephosphorylating activity towards recombinant substrates (8.6%, 2.6%, and 27.1% decrease of ERK phosphorylation for SHP-1, MKP3 and PP2A respectively and 5% decrease of p38 phosphorylation by MKP1) (**Figure 4G, 4H and 4I**). Therefore, decrease in the phosphatase activity significantly contributes in GRA-mediated MAPK activation.

### GRA modulates kinase/phosphatase balance in infected mice

**Figure 5:**
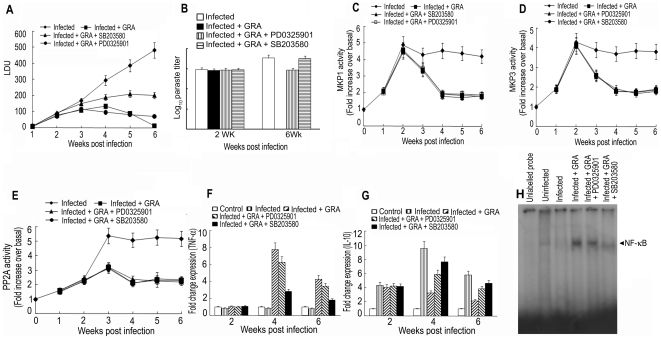
Effect of GRA on in vivo kinase/phosphatases balance, modulation of parasitemia and cytokine response. *L. donovani*-infected mice was treated with GRA (50 mg/kg) i.p. for 3 times 5 days apart starting at 10 days post-infection. In a separate group, infected mice were treated with either p38 inhibitor SB203580-HCL (10 mg/kg) or ERK-inhibitor PD0325901 (20 mg/kg) over a 4 wk period along with GRA. (**A)** Spleen parasite burdens were determined weekly in each group and expressed as LDU ± SD. (**B**) Spleen parasite burden determined 2 and 6 wk following infection measured by limiting dilution assay expressed as log_10_ parasite burden ± SD. MKP1, MKP3 and PP2A were immunoprecipitated from cell lysates of splenocytes isolated from different experimental groups with respective antibodies. MKP1 (**C**) and MKP3 (**D**) and PP2A (**E**) activity was assayed by pNPP hydrolysis and assay kit respectively. Expression of TNF-α (**F**) and IL-10 (**G**) at mRNA level were determined by Real time PCR in the splenocytes. (**H**) NF-κB DNA binding activity was determined in the splenocytes of different experimental groups as described in the legend of **Figure 1B**. Animal experiments were done with five animals/group and the results are representative of three individual experiments. ns, not significant; **p<0.01, ***p<0.001; Student's t-test.

In order to ascertain the role of GRA on cellular kinase/phosphatase balance *in vivo*, GRA (50 mg/kg/day) was administered i.p. in *L. donovani*-infected mice for 3 times 5 days apart starting at 10 days post-infection and splenocytes were isolated. To gain insight into the effect of p38 and ERK pathways in GRA-mediated curative response, separate groups of infected mice were treated with p38 inhibitor SB203580-HCL (10 mg/kg; twice weekly) or ERK-inhibitor PD0325901 (20 mg/kg; twice weekly) over a 4-wk period along with GRA. In all the four groups (infected, infected GRA-treated, infected GRA-treated as well as p38 inhibitor-treated and infected GRA-treated as well as ERK1/2 inhibitor-treated), spleen parasite burden and phosphatase (MKP1, MKP3 and PP2A) activities were measured. Similar to the *in vitro* scenario, in infected animals, activities of all three phosphatases were elevated, being maximum at 2 wk for MKPs (4.9-fold for MKP1 and 4.3-fold for MKP3) and at 3 wk for PP2A (5.4-fold). For all three phosphatases, enzymatic activity was found to be elevated as studied up to 6 wk of infection (**Figure 5C, 5D and 5E**). On the contrary, GRA-treated mice showed almost complete suppression of spleen parasite burden at 6 wk (**Figure 5A and 5B**) along with a substantial decrease of MKP1 (75.4%), MKP3 (68.2%) and PP2A (69.3%) activities (**Figure 5C, 5D and 5E**). GRA-mediated suppression of spleen parasite burden was significantly reversed in p38 inhibitor-treated mice whereas the effect was much less in ERK inhibitor-treated animals (**Figure 5A and 5B**). Interestingly, *in vivo* pharmacologic inhibition of p38 and ERK pathway could not alter GRA-mediated inhibition of phosphatase activities, which suggested that GRA-mediated anti-leishmanial effect is dependent on both kinase and phosphatase activities. Down regulation of phosphatase activity alone is not enough to vouch for GRA's immunomodulatory property. To further substantiate the importance of MAPK pathway in GRA-mediated curative effect, we checked whether GRA-mediated modulation of pro/anti-inflammatory balance were altered in presence of MAPK inhibitors. GRA treatment resulted in increased mRNA expression of TNF-α (8.9-fold) (**Figure 5F**) along with a concomitant decrease (74.1%) in IL-10 expression (**Figure 5G**) in infected animals at 4 wk. On the contrary, administration of p38 inhibitor markedly attenuated GRA-induced TNF-α expression (73.1%) along with reversal of GRA-mediated suppression of IL-10 (23.2%) expression. Moreover, *in vivo* DNA binding activity of NF-κB in GRA-treated infected animal was significantly reduced in presence of p38 inhibitor although to a much lesser extent in ERK inhibitor treated animals (**Figure 5H**). These results clearly demonstrate that i) p38 MAPK activation plays a predominant role in GRA-mediated *in vivo* activation of NF-κB as well as antileishmanial effector response and ii) kinase/phosphatase balance is vital for intracellular infection and GRA could modulate this balance in favour of the host.

## Discussion

In the susceptible host, control of visceral leishmaniasis revolves around the state of activation of the tissue mononuclear phagocytes. Unstimulated resident macrophages of liver, spleen and bone marrow, the target cells of this disseminated intracellular infection, initially support parasite replication. However, if appropriately activated, the same macrophages and tissue-homing blood monocytes develop sufficient leishmanicidal activity to control and largely resolve the infection [31]. Immunomodulators are becoming popular as promising alternative to traditional medicine because they play a particularly prominent macrophage-activating role, which extends to the priming of macrophages to secrete leishmanicidal molecules that include iNOS-derived reactive nitrogen intermediates and proinflammatory cytokines [32]. GRA is one such immunomodulator of herbal origin as it is known to stimulate macrophage-derived NO production, and is able to upregulate iNOS expression through NF-κB transactivation in murine macrophages [12]. Previously we documented that GRA has strong potential to be used as a potent antileishmanial agent and this effect could be attributed to the NF-κB up-regulatory property of GRA [15]. In the present work, we tried to elucidate the intracellular signaling mechanism of GRA responsible for its antileishmanial effect. We found that amongst three major MAP kinases responsible for proinflammatory cytokine production, p38 MAPK primarily and to a lesser extent ERK contribute in GRA- mediated activation of NF-κB as well as anti-leishmanial cellular and immunological response. Interestingly, GRA-mediated MAPK activation could be an after effect of down regulation of MAPK directed phosphatases, which are activated during infection. Therefore, GRA might exert its immunomodulatory effect by modulating cellular kinase/phosphatase balance, which often plays a very important role in dictating the fate of intracellular infection [33].

The importance of NF-κB in resistance to *Leishmania* infection has been clearly established by studies in which mice deficient in different NF-κB family members were shown to be susceptible to parasitic infections [34]. We first demonstrated that, as an efficient immunomodulator, GRA activated NF-κB pathway in parasite-infected BALB/c mice. The most prevalent activated form p65 and p50 contribute mainly in GRA- mediated activation of NF-κB in *in vivo*. Furthermore, the results provide the first *in vivo* evidence that GRA may exert its curative effects on experimental visceral leishmaniasis mostly through its ability to activate NF-κB by inducing phosphorylation and subsequent degradation of IκBα. Using pharmacologic inhibitor NF-κB, we also demonstrated that activation of NF-κB is absolutely essential for GRA-mediated suppression of spleen parasite burden and induction of proinflammatory gene expression.

As MAPK pathways play a major role in proinflammatory cytokine synthesis, rigid control mechanisms exist to prevent inappropriate activation, which could be detrimental for the cell. On the other hand, some opportunistic pathogens like *Leishmania* take advantage of this regulation and use the system in favour of their own survival within macrophages. Studies revealed that *Leishmania* impair the CD40 effector functions by interrupting the CD40 signaling through p38 MAPK in macrophages [35]. NO production and killing of *L. donavani* parasite in infected macrophages in response to sodium antimony gluconate treatment involved mainly PI3K and p38 MAPK [36] and activation of p38 MAPK has also been shown to play an important role in eliminating *L. donovani* infection [37]. All these studies suggest that compounds having ability of augmenting signaling pathways leading to macrophage activation might demonstrate protective action against *Leishmania* infection. In the present study GRA enhanced NF-κB activity at both *in vitro* and *in vivo* situation which was found to be mainly under the control of the p38 pathway and to a lesser extent ERK1/2 pathway. Direct correlation between MAPK activation and upregulation of both NF-κB activity and NO production has been shown in a number of studies [16], [17], [20]. A growing body of evidences indicate that MSK1, a nuclear kinase downstream of both ERK1/2 and p38 and might therefore be an end-kinase in the inflammatory process involving NF-κB, act as a post-translational modifier of NF-κB by inducing phosphorylation of p65 subunit at Ser^276^ residue [30]. The present study revealed that GRA could also activate MSK1 in BMDM that was under the control of ERK1/2 and p38 as pharmacologic inhibition of both these MAPKs markedly attenuated GRA-induced MSK1 activity. Activation of MAPKs are tightly regulated in phagocytic cells by counteracting protein phosphatases and we have recently demonstrated that MAPK directed phosphatases play a crucial role in disease progression of visceral leishmaniasis by modulating pro/anti-inflammatory cytokine balance [27]. The role of SHP-1 in modulating visceral infection has already been documented [24], [26]. It is possible that MAPK activation by GRA could be an after-effect of down regulation of all these phosphatases. Selective dephosphorylation of recombinant p38 and ERK substrate in *L. donovani*-infected macrophages were markedly attenuated by GRA, which may be associated with down-regulation of MKP1, MKP3 and PP2A expression. A different scenario was obtained for SHP-1, where *L. donovani* infection only increased the activity without altering its expression level. GRA treatment decreased the activity of SHP-1 as evidenced by ERK dephosphorylation assay. p38, the major MAPK activated by GRA, was not regulated by SHP-1, which is in accordance with previous reportss [38]. In the *in vivo* situation also, GRA treatment markedly abrogated MKP1, MKP3 and PP2A activity in *L. donovani*-infected mice. Interestingly, although *in vivo* blockage of p38 and ERK pathway could not alter GRA-mediated inhibition of phosphatase activities but these could reverse GRA-induced suppression of spleen parasite burden and IL-10 synthesis as well as induction of TNF-α synthesis. Regulation of either kinases or phosphatases by GRA is not the deciding factor for the outcome of infection in VL. It is the overall shifting of kinase/phosphatases balance towards kinases, which is important to exert curative responses by GRA.

GRA, therefore, could be a new class of macrophage activators to stimulate the induction of iNOS and pro-inflammatory cytokines. It is used as a natural food ingredient and as flavouring and sweetening agent in food products. It has also been widely used as an antidote, demulcent and as a folk medicine for generations in Asia and Europe. A dose of 50 mg/kg body weight of GRA could completely eliminate the spleen parasite burden in experimental mouse model of VL. In extrapolating this dose to an 80-kg human with a surface area of 2 m^2^ (Costeff's rule), it would take a total dose of 4 g (50 mg×80) or 2 g/m^2^ of surface area, which is within the nontoxic consumption limit of GRA [39]. Overall, a comprehensive understanding of the detailed upstream signaling events by which GRA triggers curative response against experimental visceral leishmaniasis could better define its potential as an effective immunomodulator not only for non healing leishmaniasis but also for other chronic diseases.

## Materials and Methods

### Ethics statement

This study was carried out in strict accordance with the recommendations in the Guide for the Care and Use of Laboratory Animals of the National Institutes of Health. The protocol was approved by the Committee on the Ethics of Animal Experiments of Indian Institute of Chemical Biology (Permit Number: 147-1999).

### Parasites, cell culture, NO production and infection

*L. donovani* promastigotes ((MHOM/IN/1983/AG83) were grown as described earlier [40]. Peritoneal macrophages and BMDM from BALB/c mice were isolated and maintained as described earlier [15], [27]. For BMDM, bone marrow cells were isolated from the femurs and tibias of 6–8 wk old BALB/c mice and were suspended in RPMI 1640 supplemented with 100 U/ml penicillin and 100 µg/ml streptomycin, 10% FCS, 5% horse serum, and 20% L929-conditioned media and were incubated at 5% CO_2_, 95% humidity, at 37°C. On the 4th day, cells were fed again with L-cell conditioned medium; on day 6, cells were harvested, counted and used for experiments. Splenocytes were cultured as described previously [40]. *In vitro* infection experiments were carried out at a 10∶1 parasite/cell ratio as described earlier [27]. The numbers of infected macrophages (% of infection) and parasite burden (number of parasites per infected macrophages) were determined by counting 200 macrophages. Nitrite formation in macrophage culture supernatants was detected by the Griess reaction as previously described [15]. For *in vivo* experiments, female BALB/c mice were injected via the tail vein with 10^7^ *L. donovani* promastigotes. For reinfection experiments, promastigotes were injected 8 wk after the first infection. GRA (Sigma Aldrich) (10–100 mg/kg/day) was administered i.p. for 3 times 5 days apart starting at 10 days post-infection. Infection was assessed by removing spleen from infected mice at different time periods and parasite burdens were determined from Giemsa-stained impression smears. Data are presented as Leishman–Donovan units (LDU) [41]. To further ascertain whether the spleen contained live parasites, in selected experiments, the parasite burden in spleen was also quantified by limiting dilution technique according to Banerjee et al. [42]. In brief, a measured amount of spleen was homogenized in Schneider's medium (Invitrogen) supplemented with 10% FCS and diluted to a final concentration of 1 mg/ml. This was then serially diluted up to five fold and cultured at 22°C for 14 days. Reciprocal of the highest dilution positive for live parasites was considered to be the concentration of parasites per milligram of spleen and spleen parasite burden was calculated from the total weight of the spleen.

### Real-time PCR

Total RNA from cells was isolated using the RNeasy mini kit (Qiagen) according to the manufacturer's instructions. cDNA was synthesized from 1 µg of DNA using the SuperScript first strand synthesis system for the RT-PCR kit (Invitrogen). TaqMan probes for IL-12, TNF-α and iNOS were purchased from Applied Biosystems. Quantitative real-time PCRs were performed in the ABI 7500 Fast Sequence detector with the following PCR amplification conditions: 40 cycles of 95°C for 15 s and 60°C for 1 min. Relative quantitation was performed using the comparative ΔΔ*C*t method and data was normalized to mouse β-actin mRNA levels and expressed as a –fold change compared to uninfected controls.

### Western Blotting and ELISA

Immunoblotting was performed as previously described [15], [27]. Densitometric analyses for all experiments were carried out using QUANTITY ONE software (Bio-Rad). Band intensities for immunoblots were normalized to β-actin and expressed in arbitrary units. For ELISA, the level of various cytokines in the culture supernatants were measured using a sandwich ELISA kit (Quantikine M, R&D Systems) as per the detailed instructions of the manufacturer.

### MSK1 activity assay

MSK1 assay was performed as described previously [20]. Briefly, treated macrophages were washed with ice-cold PBS and lysed in lysis buffer containing 50 mM Tris-HCl, pH 7.5, 1 mM EDTA, 1 mM EGTA, 1 mM sodium orthovanadate, 1% (v/v) Triton X-100, 50 mM NaF, 5 mM sodium pyrophosphate, 0.27 M sucrose, 0.1%(v/v) β mercaptoethanol, 1 mM benzamidine, 2 µg/ml leupeptin, and 0.2 mM pefabloc. Whole cell lysates (1 mg) collected by centrifugation (5 min, 10,000× g, 4°C), were immunoprecipitated with 2 µg of anti-MSK1 Ab for 2 h at 4°C. Protein G–agarose was added and further incubated for 2 h. After centrifugation, the pellets were washed twice with cell lysis buffer and twice with kinase assay buffer (Cell Signaling Technology). Immunoprecipitated MSK1 pellets were incubated at 30°C for 30 min in 50 µl of kinase buffer containing 30 µM substrate peptide: EILSRRPSYRK (CREBTIDE) and 0.1 mM [γ^32^P] ATP (200,000 cpm/pmol). Reactions were stopped by placing the tubes on ice. After centrifugation (30 s, 4°C, 10000× g), 30 µl of supernatant was spotted on p81 phosphocellulose paper, washed thrice with 0.75 M orthophosphoric acid and incorporation of radiolabelled phosphates in CREBTIDE was determined.

### IκB kinase β (IKKβ) assay

IKKβ assay was performed as described earlier [20]. Subcellular protein fractions were prepared from freshly isolated spleen in cold kinase assay lysis buffer (20 mM Tris-HCl, pH 8.0, 500 mM NaCl, 1 mM EDTA, 1 mM EGTA, 10 mM β-glycerophosphate, 10 mM NaF, 10 mM pNPP, 300 µM Na_3_VO_4_, 1 mM benzamidine, 2 µM PMSF, 10 µg/ml aprotinin, 1 µg/ml leupeptin, 1 µg/ml pepstatin, 1 mM DTT and 0.25% Nonidet P-40) for IKK activity. The IKK activity was measured as described earlier with slight modification [15]. Briefly, the cell lysates (500 µg) were immunoprecipitated with anti-IKKβ Ab in immunoprecipitation buffer [25] and immunoprecipitated samples were incubated with recombinant IκBα (4 µg) in kinase buffer (Cell Signaling Technology) at 30°C for 1 h. The kinase reaction was stopped by addition of SDS-sample buffer. The sample was resolved by SDS-PAGE, dried and autoradiographed. To determine the total amount of IKKβ in each sample, 30 µg of the whole cell extract protein was subjected to SDS-PAGE and analyzed by Western blot using anti-IKKβ Ab.

### Electrophoretic mobility shift assay (EMSA)

EMSA was performed in isolated splenocytes as described earlier [20]. Briefly, isolated spleen cells were resuspended in hypotonic buffer (10 mM HEPES, pH 7.9, 1.5 mM MgCl_2_, 10 mM KCl, 0.2 mM PMSF and 0.5 mM DTT), and allowed to swell on ice for 10 min and treated with 1% NP-40. Cells were homogenized in a Dounce homogenizer. The nuclei were separated by spinning at 3,300× g for 5 min at 4°C. The nuclear pellet was extracted in nuclear extraction buffer (20 mM HEPES, pH 7.9, 0.4 M NaCl, 1.5 mM MgCl_2_, 0.2 mM EDTA, 25% glycerol, 0.5 mM PMSF and 0.5 mM DTT) for 30 min on ice, and centrifuged at 12,000× g for 30 min. Supernatants containing the nuclear extract were collected and used for EMSA. For the preparation of radiolabeled probes representing standard consensus sequences of NF-κB, the following oligonucleotides were used: 5′-AGT TGA GGG GAC TTT CCC AGG C-3′. As a control, a 100-fold molar excess of unlabelled competitor oligonucleotide was added. For supershift assay, the nuclear extracts were incubated with Ab against individual components of NF-κB complex (Santa Cruz Biotechnology) for 30 min at 25°C and analyzed by EMSA in the presence of all components of the binding reaction described earlier [15].

### Transient transfection and reporter assay

Transfections and NF-κB luciferase activity were performed as described previously [20]. Transfections were carried out in 2×10^6^ cells with the appropriate constructs in serum free medium using Lipofectamine (Invitrogen Life Technologies) according to the manufacturers' instruction. Three hour after transfection, the cells were washed and replaced with RPMI medium containing 10% FCS. After 24 h, cells were processed as indicated in figure legend. For NF-κB luciferase activity, cells were harvested using reporter lysis buffer (Promega) and luciferase activity was then assessed via luminometry. The value of luciferase activity was normalized to transfection efficiency monitored by the co-transfected β-galactosidase expression vector.

### Fluorescence microscopy

Macrophages (5×10^5^) were plated onto 18 mm^2^ coverslips kept in 30 mm petri plates and cultured overnight. The cells were then incubated with *L. donovani* promastigotes, both alone or in combination with GRA (20 µM) for 3 h, washed twice in PBS, and fixed with methanol for 15 min at room temperature. After fixation the cells were permeablized with 0.1% Triton X and incubated with NF-κB p65 antibody for 1 h at 4°C. After washing, coverslips were incubated with allophycocyanin (APC)-conjugated secondary antibody (1 h, 4°C). The cells were then stained with DAPI (4′,6-diamidino-2-phenylindole, 1 µg/ml) in PBS plus 10 µg/ml Rnase A to label the nucleus, mounted on slides and visualized under Olympus BX61 microscope at a magnification of 1000 and the images thus captured were processed using ImagePro Plus (Media Cybernetics).

### PTP activity assay

PTP activity was measured using PTP Assay kit (Sigma-Aldrich) as described earlier [27]. Briefly, infected and GRA-treated BMDM were lysed in lysis buffer (50 mM Hepes, pH 7.4, containing 0.5% Triton X-100, 10% glycerol, 1 mM benzamidine, 10 µg/ml aprotinin, 10 µg/ml leupeptin, and 2 µg/ml pepstatin A), incubated on ice for 30 min and centrifuged at 10,000× g for 15 min at 4°C. Protein content was determined in cleared lysate by Bio-Rad protein assay. 10 µg of protein extract were used to measure PTP activity using monophosphorylated phosphotyrosine peptide as substrate. Free inorganic phosphate was detected with malachite green (Sigma-Aldrich), and OD was taken at 620 nm.

### Ex vivo phosphatase assay

Enzyme activity for MKP1, MKP3 and PP2A were determined from isolated splenocytes as described previously [27]. Briefly, MKP1 and MKP3 were immunoprecipitated from 300 µg of the whole cell lysate using 2 µg of anti-MKP1 or anti-MKP3 antibody and 100 µl of protein A-Sepharose (Santa Cruz Biotechnology) at 4°C for 2 h. Immune complexes were collected, washed 4 times and finally resuspended in 100 µl lysis buffer. The assay was performed in phosphatases assay buffer at 37°C for 30 min. Reactions were terminated by addition of 50 µl of 200 mM NaOH and absorbance was taken at 410 nm. PP2A activity was determined as described previously [27] by using the Serine/Threonine Phosphatase Assay System from Promega.

### MAPK dephosphorylation assay

For MAPK dephosphorylation assay, whole cell protein was obtained by lysing the cells in lysis buffer [50 mM Tris-HCl, pH 7.4, 150 mM NaCl, 1% NP-40 and complete protease inhibitors (Roche Applied Science)]. Immunoprecipitation was performed by incubation of cell lysate (500 µg/500 µl) with 5 µg of respective antibodies (anti SHP-1, anti-MKP1, anti-MKP3 or anti-PP2A) and rocked overnight at 4°C. 50 µl/sample protein G plus-agarose (Santa Cruz Biotechnology) was added and rocking was allowed to continue for another 1 h. The immunoprecipitates were washed twice with lysis buffer and once with phosphatase buffer (50 mM Tris-HCl, pH 7.5, 1 mM MgCl_2_, 0.1 mM EDTA and 100 µg/ml bovine serum albumin, 50 µg/ml leupeptin) in non-reducing conditions without β-mercaptoethanol. Immunoprecipitated phosphatases were incubated with 0.1 µg of recombinant p-ERK (Stratagene) or 0.25 µg of recombinant p-p38 [43] at 37°C in phosphatase buffer [27]. The reaction was terminated by adding 2× sample buffer. The samples were separated on a SDS-PAGE and blotted with p-ERK or p-p38 antibody.

### Statistical analysis

Data shown are representative of at least three independent experiments unless otherwise stated as n values given in the legend. Macrophage cultures were set in triplicates and the results are expressed as the mean ± SD. Student's t test was employed to assess the statistical significances of differences among pair of data sets with a *p* value<0.05 considered to be significant.
